# Cationic Pullulan Derivatives Based Flocculants for Removal of Some Metal Oxides from Simulated Wastewater

**DOI:** 10.3390/ijms24054383

**Published:** 2023-02-23

**Authors:** Luminita Ghimici, Maria Magdalena Nafureanu, Marieta Constantin

**Affiliations:** “Petru Poni” Institute of Macromolecular Chemistry, Aleea Grigore Ghica Voda 41A, 700487 Iasi, Romania

**Keywords:** cationic pullulan derivatives, metal oxides, synthetic wastewater, flocculation evaluation, flocculation mechanism, wastewater treatment

## Abstract

Modified polysaccharides have been increasingly used as flocculants in wastewater treatment due to their non-toxicity, low price, biodegradability, etc. However, the pullulan derivatives are less used in wastewater purification processes. Therefore, this article presents some data regarding FeO and TiO_2_ particle removal from model suspensions by some pullulan derivatives with pendant quaternary ammonium salt groups, trimethylammonium propyl carbamate chloride (*TMAP_x_–P*). The influence of the polymer ionic content, dose, and initial solution concentration as well as of the dispersion pH and composition (metal oxide content, salts, and kaolin) on the separation efficacy were considered. UV-Vis spectroscopy measurements have shown a very good removal efficacy of *TMAP_x_–P* for the FeO particles (around 95% and more), irrespective of the polymer and suspension characteristics; a lower clarification of the TiO_2_ particles suspension (removal efficiency between 68% and 75%) was noticed. Both the zeta potential and the particle aggregates size measurements revealed the charge patch as the main mechanism which governs the metal oxide removal process. The surface morphology analysis/EDX data provided supplementary evidence regarding the separation process. A good removal efficiency (90%) of the pullulan derivatives/FeO flocs for the Bordeaux mixture particles from simulated wastewater was found.

## 1. Introduction

Pullulan, a non-ionic polysaccharide produced by some microorganisms (*Aureobasidium pullulans*, *Teloschistes flavicans*, *Cytariaharioti*, etc.) and different carbon sources (jackfruit seed, cassava bagasse, ricehull, etc.) [1], has attracted expanding interest due to the advantages encountered, generally, of the polymeric materials belonging to polysaccharides class (non-toxicity, low price, easy availability, biodegradability), but also related to its chemical structure (high flexibility of the chain, high solubility in water, ability to be modified by different chemical reactions, etc) [1,2,3]. The wide range of chemical reactions used to modify this polysaccharide (esterification, sulfation, copolymerization, and oxidation) [4,5,6,7,8,9,10] lead to pullulan derivatives that, apart from the properties of the unmodified sample, may exhibit new ones, suitable for certain applications. For example, the introduction of ionizable groups such as carboxylic, quaternary ammonium, or amino ones, which dissociate in polar solvents and lead to charged polymer chains, can give the ionic pullulan derivatives the possibility to interact with oppositely charged particles by means of the electrostatic attraction forces, thus becoming suitable polymers for using in the wastewater purification field. Microspheres of pullulan-graft-poly(3-acrylamidopropyl trimethylammonium chloride) and some soluble pullulan derivatives containing either tertiary amine groups (aminopropyl dimethylamine) or quaternary ammonium salt ones (grafted chains, poly[(3-acrylamidopropyl)-trimethylammonium chloride]) have been already tested and successfully removed some organic particles, such as dyes (*Methyl Orange, Acid Orange 7, Azocarmine B, Methylene Blue, Reactive Blue 2*) [11] and commercial pesticide formulations (*Fastac 10EC, Karate Zeon, Novadim Progress, Bordeaux mixture, Decis, Confidor oil, Confidor Energy*) [12,13] as well as inorganic ones (clay and kreutzonit) [12,14] from simulated wastewater. The latter particles are mostly used in some ceramic industry processes, namely for the sprocket gypsie and glaze preparation, and are, therefore, found in ceramic wastewater. Along with the aforementioned particles, metal oxides (iron oxides (Fe_2_O_3_, FeO), cobalt oxide (CoO), copper oxide (CuO), nickel oxide (NiO), etc.), and zinc oxide (ZnO)) are used in ceramics to impart color to glazes and bodies or as opacifier agents (titanium oxide (TiO_2_)). Many of them are also used in paints, coatings, adhesives, rubber, printing inks, cosmetics, textiles industries, etc. Their presence in wastewater which has to be reused could have undesired effects on different steps of the industrial processes mentioned above. Thus, some effort has been spent to remove them from wastewater by adsorption/flocculation methods in order to protect the water resources [15,16,17,18,19,20,21,22,23]. So far, the flocculants were based either on synthetic polymer (homopolymers and graft copolymers of acrylamide (AM) and diallyldimethylammonium chloride (DADMAC) [15,23], PEG, and Pluronic-type polymers [19] or polysaccharides derivatives based on chitosan [17,21], dextran [18], and cellulose [20]).

The inspection of the literature data reveals that until now there are no systematic data concerning the application of the pullulan derivatives as flocculants for the removal of metal oxides. The lack of this kind of information, on the one hand, and the excellent performance exhibited by the grafted pullulan derivatives as well as those with pendant tertiary amine groups in the removal of clay and kreutzonit particles (removal efficiency (RE%) between 95–99%), on the other hand, encouraged us to investigate the efficiency of some pullulan derivatives containing different amounts of pendant strong basic quaternary ammonium salt groups, trimethylammonium propyl carbamate pullulan (*TMAP_x_–P*) randomly distributed along the polymer backbone in the removal of some metal oxides, namely FeO and TiO_2_ from simulated wastewater. The results will be compared with those obtained when some of the polymers mentioned above were used as flocculants (Table 1).

Our research considered the influence of some tunable parameters, such as the pullulan derivatives ionic content, polymer dose, and initial concentration (*c*_ip_), dispersion pH and composition (contaminant content (*c*_FeO_), salts and kaolin presence), as the main parameters influencing the separation efficacy. 

UV-Vis spectroscopy, zeta potential, the particle aggregates size measurements together with surface morphology, and EDX analyses were used as tools to evaluate the flocculation efficacy of the pullulan derivatives investigated herein and the possible mechanisms controlling the metal oxides removal process.

## 2. Results and Discussion

The optimum dose (*dose*_op_) (the polymer dose (PD) corresponding to the maximum removal efficiency of contaminants) is one of the terms, routinely used to express the efficiency of the flocculation process. It can be influenced by parameters related either to the chemical structure of polymers (molecular mass, ionic group content and nature, the alkyl chain length, etc.) or dispersion medium characteristics (composition, pH, temperature, the polymer and contaminant concentration, etc.). In the following, some data regarding the influence of some of the aforementioned parameters on the *dose*_op_, and hence the separation efficacy of the pullulan derivatives investigated in dispersions of FeO and TiO_2_ particles, are presented.

### 2.1. Pullulan Derivatives Ionic Group Content

- FeO suspension

**Figure 1:**
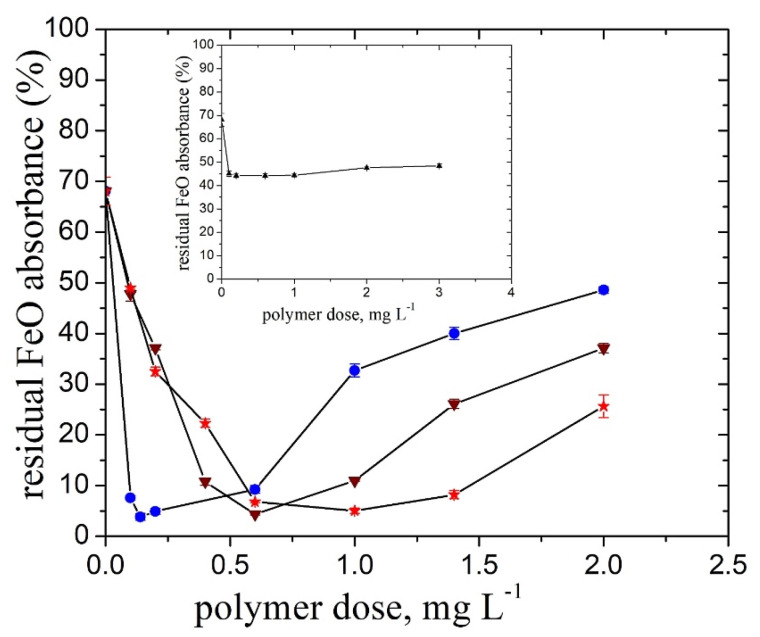
The residual FeO absorbance (%) dependence on the polymer dose for: *TMAP*_0.7_*–P* (circle); *TMAP*_0.4_*–P* (inverted triangle); *TMAP*_0.2_*–P*; (star); the inset: P (triangle); *c*_ip_ = 1 g L^−1^, *c*_FeO_ = 0.4 g L^−1^, pH = 8.34, settling time 120 min.

The ionic group content of a polyelectrolyte chain is, probably, the parameter with the greatest impact on the chain conformation in solution, which in turn, influences the flocculation ability of an ionic polymer. The impact of a series of *TMAP_x_–P* having different ionic content (see Table 3, Section 3.1. Materials) on the removal of FeO (*c*_Feo_ = 0.4 g L^−1^) and TiO_2_ (*c*_TiO2_ = 0.05 g L^−1^) particles has been examined and presented below. The residual FeO absorbance vs. *TMAP_x_−P* dose plots (Figure 1) show a very good removal of the FeO particles (removal efficiency around 95% and more) in the polymer dose interval between 0.14 mg L^−1^ (*TMAP*_0.7_*-P*) and 1 mg L^−1^ (*TMAP*_0.2_*–P*); according to the calibration curve, the residual FeO concentration is 0.014 g L^−1^ (*TMAP*_0.7_*–P*) and 0.015 g L^−1^ (*TMAP*_0.2_*–P*). 

The investigations regarding the polymer–particle interactions have brought out the diversity of forces (electrostatic attractions, hydrogen bonding, ion binding, hydrophobic ones) implied in these systems, depending on the characteristic of the partners investigated [24]. Taking into account (i) the presence of the quaternary ammonium salt groups on the *TMAP_x_–P* chains and the negatively charged FeO particles (ζ = −26.7 mV—see the Experimental Section 3) at the working pH (7.1) and (ii) the poor performance of the unmodified pullulan sample in the separation of this type of particles (maximum removal efficiency around 55%) (Figure 1, inset), one may assert that the electrostatic attraction forces between the oppositely charged sites on the *TMAP_x_–P* and FeO particles prevail in the separation process. This assumption is supported by another finding, worthy to underline, namely the decreasing of *dose*_op_ values with augmentation of the ionic group content (Table 2). 

**Figure 2:**
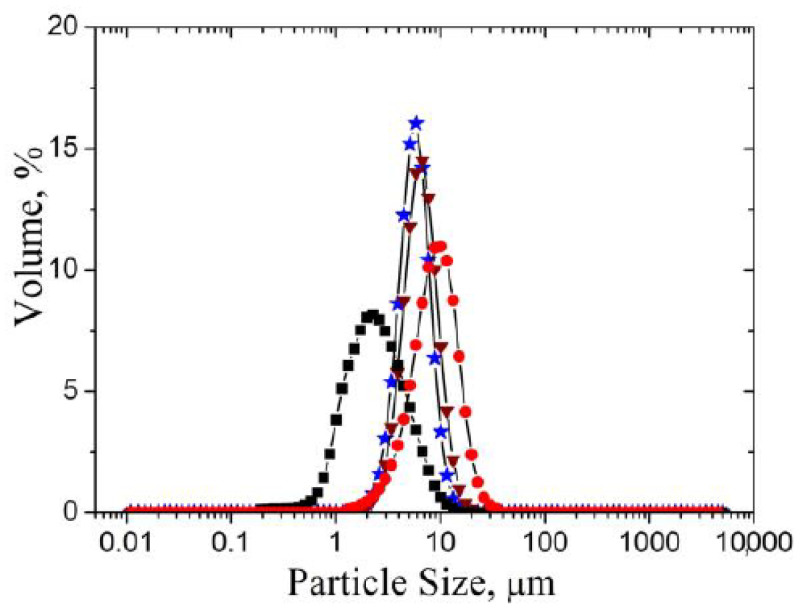
Particle size distribution for initial FeO particles (square) and aggregates obtained at *dose*_op_ as a function of the ionic group content, *TMAP*_0.7_*–P* (circle); *TMAP*_0.4_*–P* (inverted triangle); *TMAP*_0.2_*–P* (star); and settling time 120 min. The particle size distribution curves represent the mean of three independent measurements.

A higher amount of charged groups on the polymer chains means an increased ability to electrostatically attract the negative charges on the FeO particle′s surface, and hence the separation process. The neutralization process is also helped by the more extended chains of the highest charged sample (*TMAP*_0.7_*–P*), conformation resulting from the increased electrostatic repulsion between the positively charged segments of the chain. Moreover, the enhancing efficacy in FeO/*TMAP_x_–P* interactions with augmentation pullulan derivatives charged content is reflected in flocs size (median values D50, µm) recorded at *dose*_op_ for each pullulan derivative sample, which follows the next order: D50*_TMAP_*_0.2–*P*_ *=* 5.23 ± 0.16 µm *<* D50*_TMAP_*_0.4–*P*_ *=* 5.99 ± 0.33 µm < D50*_TMAP_*_0.7–*P*_ *=* 8.32 ± 0.39 µm (Figure 2).

**Figure 3:**
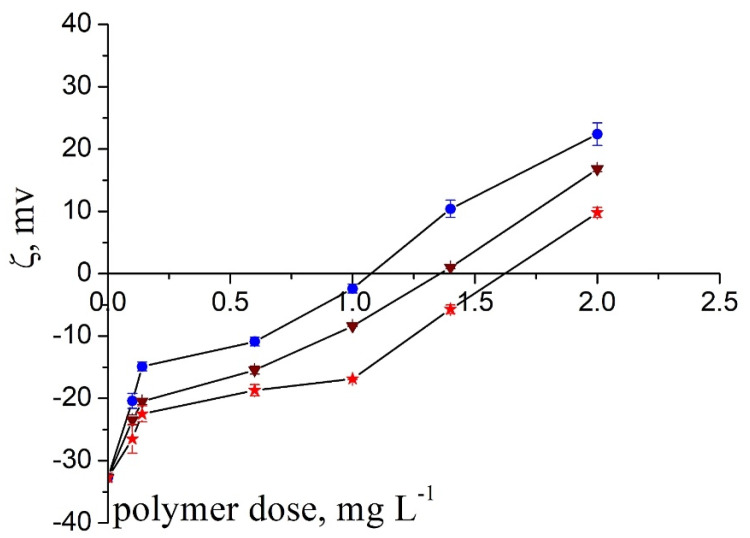
Zeta potential (ζ) dependence on the polycation dose for *TMAP*_0.7_*–P* (circle); *TMAP*_0.4_*–P* (inverted triangle); *TMAP*_0.2_*–P* (star); and settling time 120 min.

Anyway, the narrow size distribution and small size of the FeO/*TMAP_x_–P* aggregates, irrespective of the pullulan derivatives ionic group content, compared with that of the untreated FeO particles (D50*_FeO_*= 2.19 ± 0.02 µm) enforces the mechanisms based on the electrostatic attraction between the oppositely charged particles (charge neutralization or charge patch), as the main ones in the separation of the FeO particles. According to the finding of Claesson et al. [25], the flocs size increase with the ionic group content of polymer samples indicating that the charge patch mechanism prevails in the FeO particle removal. We would like to recall the reader that in this type of mechanism, aggregation occurs as a consequence of the electrostatic attraction forces between oppositely charged patches on different particles [24]; a higher number of charged groups at the adsorption site entail an enhanced electrostatic attraction between the oppositely charged patches on the surfaces of different particles. This result is in agreement with those found in the flocculation of some clay particles by two series of polyelectrolytes with different charge densities, namely (poly(diallyldimethylammonium chloride)-PDADMAC [26] and cationic dextran derivatives (D40-EtX) [27]. Another argument in favor of this mechanism was obtained by corroborating the UV-Vis spectroscopy results with those provided by zeta potential measurements (ζ), performed, also, in dependence of the *TMAP_x_–P* dose. As seen in Figure 3, ζ values corresponding to *dose*_op_ are negative for all of the polymer samples (−14.9 mV (*TMAP*_0.7_*–P*), −15.46 mV (*TMAP*_0.4_*–P*), and −16.86 mV (*TMAP*_0.2_*–P*)) that pleads for the charge patch mechanism, where zeta potential needs not to be zero [28], as for the neutralization mechanism [29]. 

**Figure 4:**
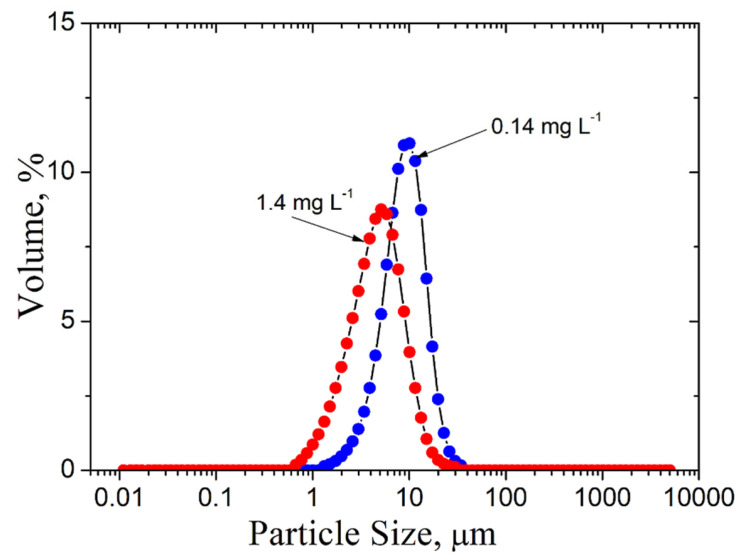
Particle size distribution for FeO/*TMAP*_0.7_*–P* aggregates at *dose*_op_: 0.14 mg L^−1^ (red circle) and polymer dose: 1.4 mg L^−1^ (blue circle), settling time 120 min. The particle size distribution curves represent the mean value of the three independent measurements.

One also observes that ζ increases for polymer doses higher than *dose*_op_ up to positive values, namely 22.4 mV in the presence of *TMAP*_0.7_*–P*, 16.8 mV for *TMAP*_0.4_*–P,* and 9.81 mV for *TMAP*_0.2_*–P*. The charge inversion of the particles and hence, their electrostatic repulsion could explain the restabilization of the FeO suspension, noticed in the UV-Vis spectroscopy measurement results (Figure 1). The inefficiency of the flocculation process at polymer doses higher than *dose*_op_ was also confirmed in the case of *TMAP*_0.7_*–P* by particle size distribution measurements (Figure 4). 

The smaller *FeO/TMAP*_0.7_*–P* aggregates size, D50 = 5.36 ± 0.17 µm, was noticed for the higher pullulan derivative dose (1.4 mg L^−1^) than that recorded at *dose*_op_, D50 *=* 8.32 ± 0.39 µm (*dose*_op_ = 0.14 mg L^−1^) might be a consequence of a surplus of positively charged polymer chains adsorbed onto the FeO particles surface; repulsion between the positive charges on the microflocs prevents their growth in larger ones.

b. TiO_2_ suspension

**Figure 5:**
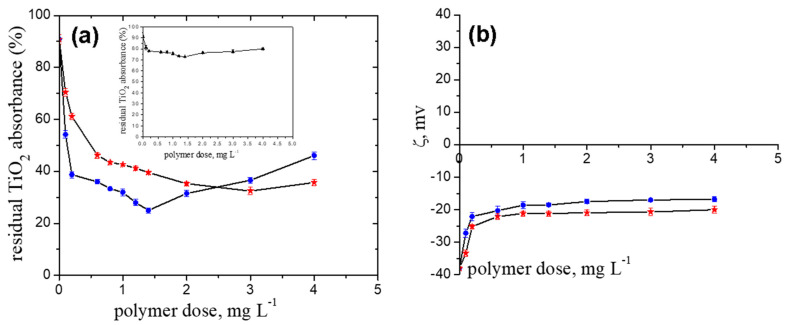
The residual TiO_2_ absorbance (%) (**a**) and zeta potential (**b**) dependence on the polymer dose for: *TMAP*_0.7_*–P* (circle); *TMAP*_0.2_*–P*; (star); the inset: P (triangle); *c*_ip_ = 1 g L^−1^, *c*_TiO2_ = 0.05 g L^−1^, pH = 5, settling time 1200 min.

In the case of the TiO_2_ particles, the ionic content influence on the particle’s flocculation resembles that noticed for the FeO particles. Therefore, only the separation performances of *TMAP*_0.2_*–P* and *TMAP*_0.7_*–P* are depicted in Figure 5a. The minimum residual TiO_2_ absorbance values were located only around 32% for the lowest charged sample (*TMAP*_0.2_*–P*) and 25% for the highest charged one (*TMAP*_0.7_*–P*) at *dose*_op_ values which increased with the ionic group content (see Table 3, Section 3.1. Materials).

The poor separation performance could be an outcome of the weak non-electrostatic and electrostatic attractive forces between the pullulan derivatives and TiO_2_ particles. The former kind of force was checked in an experiment where the unmodified pullulan sample has been used as a flocculant (Figure 5a, inset). The minimum residual TiO_2_ absorbance was 73%. This means that the attractive electrostatic interactions between the negative charges on the TiO_2_ particle surface (TiO^−^) (ζ = −38.1 mV) and the cationic groups on the polymeric chains play the most important role in the TiO_2_ particle separation. However, they are quite weak, as mentioned above, a finding also revealed by zeta potential measurements (Figure 5b). For both of the *TMAP_x_–P* samples, the increase in ζ values was small compared to the initial value (ζ_TiO2_ = −38.1 mV), over the entire range of the pullulan derivative doses investigated (up to ζ = −20 mV for the *TMAP*_0.2_*–P* and ζ = −16.8 mV for the *TMAP*_0.7_*–P*). The electrostatic repulsion interactions between the positively charged pullulan derivative segments adsorbed on different TiO_2_ particles and/or the steric ones (between the uncharged segments on the polyelectrolyte chains extended into solution, as tails, loops) could, probably, obstruct, to some measure, the polyelectrolytes/TiO_2_ particles interactions. However, one has also to remark that the pullulan derivatives efficacy in the removal of this oxide from dispersion prepared in water was higher than those of some cationic dextran derivatives (hydrophilic and amphiphilic)—removal efficiency values between 50% and 60% [17]—and PEG and the Pluronic-type polymers—removal efficiency values 56% and 20%, respectively [19] (see Table 1). Previously, Divakaran and Pillai [18] found that chitosan did not clarify a suspension of TiO_2_ particles prepared in distilled water but only in tap water; the authors surmised that some species present in the tap water played an important role in the flocculation of TiO_2_ particles. 

From the data presented above, the pullulan derivatives proved to be better flocculants for the FeO particles than for the TiO_2_ ones; therefore, the impact of some other parameters, namely *c*_ip_, dispersion pH, and composition (*c*_FeO_, salts, and kaolin presence) on their removal, was further investigated and presented below. As the impact of these parameters on the flocculation efficacy was similar for all of the three pullulan derivatives (data not shown), only one polymer sample was chosen as an example: *TMAP*_0.4_*–P* for the *c*_ip_ influence and *TMAP*_0.7_*–P* for the influence of the other parameters.

### 2.2. Initial Solution Concentration of the Pullulan Derivatives (*c*_ip_) and FeO Dispersion (c_FeO_) 

The initial solution concentration of the pullulan derivatives, *c*_ip_, refers to the concentration of the polyelectrolyte stock solution added to the clay suspension. The polymer chains conformation may vary as a function of concentration regimes, from the extended (rigid) (dilute regime) to the overlapped or even entangled one (semidilute regime) which could lead to modification of some properties, including the separation one. To find out the impact of this parameter on the FeO particle removal, flocculation tests have been undertaken with solutions of *TMAP*_0.4_*–P* with three different initial polymer concentrations, namely *c*_ip_ = 0.1 g L^−1^, 0.3 g L^−1^, and 1 g L^−1^. They are located below, close to, and above the critical overlap concentration, c* = 0.35 g L^−1^, which delimits the diluted and semi-diluted concentration regimes. c* was estimated by means of Equation (1) [30]:

c* = 1/[η](1)

where, [η] = intrinsic viscosity obtained by the Wolf method [31]; [η] = 2.81 L g^−1^.

**Figure 6:**
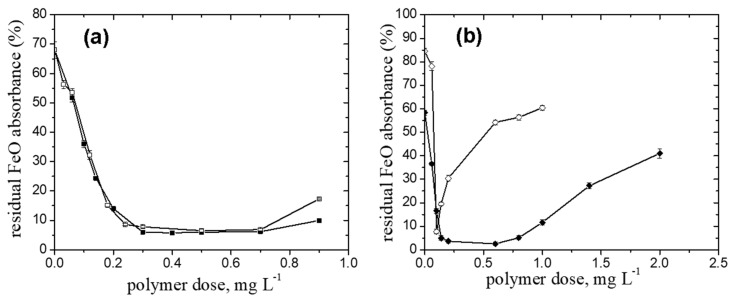
The residual FeO absorbance (%) dependence on the polymer dose: *TMAP*_0.4_*–P* for *c*_ip_ = 0.1 g L^−1^ (empty square) and *c*_ip_ = 0.3 g L^−1^ (solid square); *c*_FeO_ = 0.4 g L^−1^ (**a**) and *TMAP*_0.7_*–P* for *c*_FeO_ = 0.2 g L^−1^ (empty diamond) and *c*_FeO_ = 0.6 g L^−1^ (solid diamond); *c*_ip_ = 1 g L^−1^ (**b**); pH = 8.34, settling time 120 min.

The curves exhibited in Figure 6a (*c*_ip_ = 0.1 g L^−1^ and 0.3 g L^−1^) and Figure 1 (*c*_ip_ = 1 g L^−1^) show the removal efficiency values were quite close, between 93.5% (*c*_ip_ = 0.1 g L^−1^) and 95.66% (*c*_ip_ = 1 g L^−1^).

However, the *dose*_op_ values were different for the three *c*_ip_ investigated. They are higher for *c*_ip_ located either in the dilute regime (*c*_ip_ = 0.1 g L^−1^) or in the semidilute one (*c*_ip_ = 1 g L^−1^), *dose*_op_*=* 0.5 mg L^−1^ and *dose*_op_ = 0.6 mg L^−1^, respectively, than that recorded for *c*_ip_ close to c* (*dose*_op_ *=* 0.3 mg L^−1^). Probably, at this concentration, the polymer chains have a better arrangement on the FeO particle′s surface than in the other two cases, making easier the electrostatic attractions between the oppositely charged groups of the polymer chains and FeO particles, improving, thus, the flocculation process. One has to underline here, that this result, namely the dependence of *dose*_op_ on *c*_ip_ is different from that obtained when hydrophilic ionic polymers based on dextran were used for the flocculation of some clay particles [27]—the *dose*_op_ values were almost the same irrespective of *c*_ip_; only the residual turbidity corresponding to *dose*_op_ varied. 

These investigations revealed clearly the *c*_ip_ influence on the FeO particles removal process showing that a suitable concentration of the initial solution of *TMAP*_0.4_*–P* is required to get the best contaminant removal: *c*_ip_ = 0.3 g L^−1^ in the flocculation processes where low polymer doses are required while *c*_ip_ = 1 g L^−1^ is more suitable when a higher removal efficiency is preferred.

As regards the impact of the FeO particles concentration (*c*_FeO_) on the flocculation efficiency of the pullulan derivatives, in addition to *c*_FeO_ = 0.4 g L^−1^ (Figure 1), experiments on FeO particles suspensions with *c*_FeO_ = 0.2 g L^−1^ and 0.6 g L^−1^ at the natural suspensions pH were performed; for this test, the *TMAP*_0.7_*–P* sample was used as a flocculant (Figure 6b). 

These figures point out a negligible influence of *c*_FeO_ on the *dose*_op_ when suspensions with *c*_FeO_ = 0.2 g L^−1^ and 0.4 g L^−1^ were used, namely 0.1 mg L^−1^ in case of the former concentration and 0.14 mg L^−1^ for the latter one. A notable influence of *c*_FeO_ on the *dose*_op_ was observed for the highest *c*_FeO_ when a removal efficiency of around 95% and more was obtained for an interval of *dose*_op_ between 0.14 mg L^−1^ and 0.8 mg L^−1^. This, probably, occurs because a greater content of FeO particles in dispersion requires a higher *TMAP*_0.7_*–P* chains amount for neutralization and aggregation, hence the increase in *dose*_op_.

### 2.3. Suspension pH

**Figure 7:**
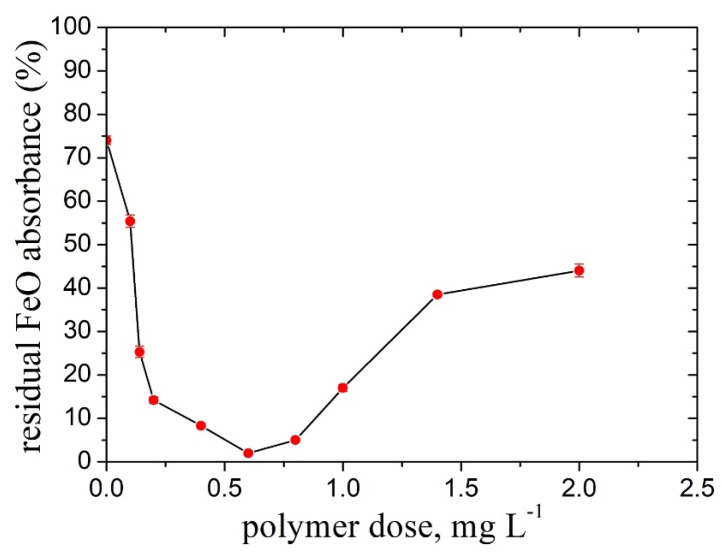
The residual FeO absorbance (%) dependence on the polymer dose (*TMAP*_0.7_*–P*) at pH 9.5, c_FeO_ = 0.4 g L^−1^, *c*_ip_ = 1 g L^−1^, settling time 120 min.

It is well known that in real wastewater, the suspension pH could change and influence, thus, the flocculation process as it can affect the charge content on the polymer chains (hence, their conformation) or particle surface. To find out the influence of this parameter on the flocculation efficacy, the experimental tests were carried out using the *TMAP*_0.7_*–P* sample on suspension of FeO particles with pH 9.5, and the results (Figure 7) were compared to those obtained on the initial suspension of FeO particles with pH 7.1 (Figure 1). The suspension pH was adjusted using 0.1M NaOH solution. The investigation of the pullulan derivative in the removal of the FeO particles at lower pH values than the natural one has not led to consistent results due to the instability of the FeO particles.

**Figure 8:**
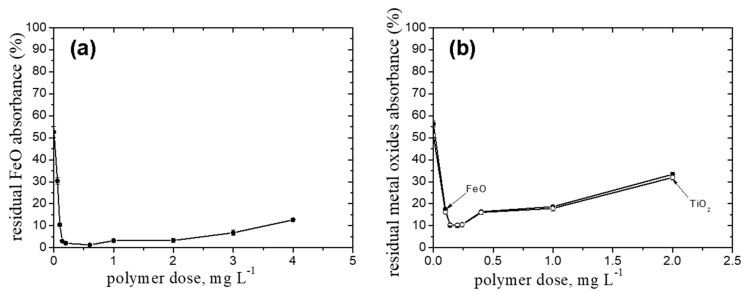
The residual metal oxides absorbance dependence (%) on the polymer dose (*TMAP*_0.7_*–P*)—suspension prepared with a mixture of FeO, NaCl, Na_2_SO_4_, NaNO_3_, CaCl_2_, MgCl_2_ (*c*_s_ = 1 × 10^−3^ M) and kaolin (0.2 g L^−1^); *c*_ip_ = 1 g L^−1^; *c*_FeO_ = 0.2 g L^−1^; settling time: 30 min (**a**) and a mixture of metal oxides (*c*_Feo_ = 0.05 g L^−1^_,_ *c*_TiO2_ = 0.05 g L^−1^), *c*_ip_ = 1 g L^−1^; settling time: 120 min (**b**).

*TMAP*_0.7_*–P* (like all the *TMAP_x_–P* samples) is a strong polyelectrolyte which means it is completely ionized irrespective of the pH value and consequently, its conformation does not depend on this parameter. As regards the FeO particles, a small increase in the medium pH from the natural one, pH 8.34 to pH 9.5, determined an increase in ζ values from −26.7 mV to −47.6 mV, which indicated an increase in the (FeO^−^) groups concentration on the oxide particles surface against that at natural pH. As seen in Figure 8, a slight enhancement of the removal efficacy (98%) and a pronounced one of the *dose*_op_ (6 mg L^−1^) were recorded; this could be explained by the stronger electrostatic attraction interactions between the FeO particles and the positive groups of *TMAP*_0.7_*–P*. This result shows that the pullulan derivative sample could be successfully used for the removal of FeO particles at a higher pH value than the natural one. 

### 2.4. FeO Particles in Suspensions Containing Salts, Kaolin, or TiO_2_ Particles

The real wastewater may contain low molecular salts, clay particles, mixtures of oxides, dyes, etc., which could influence, in a certain extent, the flocculation efficacy of a polymer. In order to evaluate the influence of other contaminants on the FeO removal percent by the pullulan derivatives investigated here, experiments on suspensions containing along with FeO particles (0.2 g L^−1^), different salts (NaCl, Na_2_SO_4_, NaNO_3_, CaCl_2_, MgCl_2_) (each salt with *c*_s_ = 1 × 10^−3^ M) and kaolin (0.2 g L^−1^) or TiO_2_ particles (FeO/TiO_2_ (*w*/*w*, 1/1)) were carried out. 

As can be seen in Figure 8a, the presence of salts and kaolin particles in the FeO suspension has not decreased the separation performance of *TMAP*_0.7_*–P*, the residual FeO absorbance being very low, between 1.31% and 3.33%, at polymer doses located in the interval 0.14 mg L^−1^ and 2 mg L^−1^, after a settling time of 30 min. 

In order to see the effects of salts or kaolin particles on the FeO particle suspensions stability, two kinds of suspensions have been prepared: one containing FeO particles and salts and the other one, FeO and kaolin particles. The residual FeO absorbance declined to about 52.66% in the former case and 93% in the latter one, after the same settling time. This means that even though the residual FeO absorbance decrease is much lower than in the presence of *TMAP*_0.7_*–P*, the salts in suspension play a certain role in the separation process.

As both metal oxides could be found in wastewater (for example in the ceramic ones), the flocculation performance of *TMAP*_0.7_*–P* (the sample with the smallest *dose_op_* used for both metal oxides) on suspensions containing mixtures of FeO/TiO_2_ (*w*/*w*, 1/1) has been checked (Figure 8b). It turned out that the polymer sample was a very good separation agent for the suspension containing the mixture of FeO and TiO_2_, a removal efficiency of about 90% of both metal oxides being noticed in the interval of polymer doses between 0.14 mg L^−1^ and 0.24 mg L^−1^ after 120 min settling time. It seems that the presence of the FeO particles helps the polymer to remove TiO_2_ particles; in the absence of *TMAP*_0.7_*–P*, the residual absorbance values of the two oxides in the mixture were 56.28% (FeO) and 51.13% (TiO_2_), after the same settling time. 

**Figure 9:**
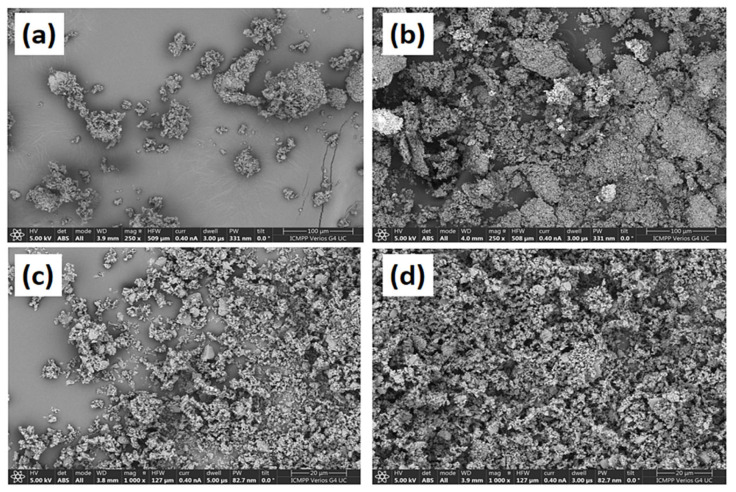
SEM micrographs of the mixtures FeO, different salts (NaCl, Na_2_SO_4_, NaNO_3_, CaCl_2_, MgCl_2_) (*c*_s_ = 1 × 10^−3^ M), kaolin (0.2 g L^−1^), and FeO and TiO_2_ (*w*/*w*, 1/1) in the absence of *TMAP*_0.7_*–P* (**a**,**c**) and their aggregates with *TMAP*_0.7_*–P* (**b**,**d**); *dose*_op_: 0.6 mg L^−1^ and *dose*_op_: 0.2 mg L^−1^, respectively.

Additional evidence for the role played by the polymer in the separation of the above mixtures was obtained with the help of the particle′s surface morphology (Figure 9a–d).

The scanning electron micrographs reveal that, in the absence of *TMAP*_0.7_*–P*, both mixtures contain separated particles of different sizes and shapes (Figure 9a,c), while bigger aggregates are formed after the interactions with polycation chains (*dose*_op_: 0.6 mg L^−1^ and *dose*_op_: 0.2 mg L^−1^, respectively) (Figure 9b,d), proving once more the role of *TMAP*_0.7_*–P* in the particles aggregation and separation. 

### 2.5. Effect of the Pullulan Derivative/FeO Flocs on the Reduction of the Fungicide Bordeaux Mixture Particles Content from Synthetic Wastewater 

It is well known that the flocculation process generates different types of sludge that, apart from a large amount of water, could contain the removed contaminants, the coagulants (aluminum- or iron-based salts)/flocculants (polymers), etc., depending on the composition of the wastewater treated and type of the treatment method applied. These, in their turn, could contaminate the groundwater, the land, etc., challenging, thus, the scientific community to find solutions for proper wastewater sludge management (disposal, regeneration, reuse). As some of them are expensive or difficult to apply (landfills, incineration, compression into building blocks, digestion) [32,33,34,35], some other cost-effective and environmentally friendly options have been investigated. Thus, the use of sewage sludge and that generated by various wastewater treatment plants, as adsorbents, has become an alternative to chemicals to remove heavy metals, phosphorus/phosphates, dyes, phenolic compounds, pesticides, etc. [36,37,38,39,40]. Regarding pesticides, one of the most dangerous types of contaminants for the whole environment (groundwater and surface water, atmosphere, soil), fertilizer and steel industry wastes for removal of 2,4-D and carbofuran pesticides [41], alum sludge for removal of glyphosate [42], activated sludge followed by pine bark adsorption for lindane and heptachlor [43], etc., have been used. Quite recently, in a preliminary test, some flocs, obtained in the flocculation process of zirconium silicate (kreutzonit) particles by grafted cationic derivatives based on pullulan, exhibited remarkable efficacy in the removal of the Bordeaux mixture particles, a fungicide widely used to combat diseases of fruits, grapes, ornamental plants, etc., from simulated wastewater; a removal efficiency higher than 95% (residual *BM* content of 25 mg L^−1^) at an optimum amount of flocs (10 g flocs/1000 mL *BM* suspension) was found [14]. Both this result and the lack of information regarding the use of polymer/contaminant flocs, as adsorbents for pesticides, motivated us to also test the ability of the FeO*/TMAP_x_–P* flocs to reduce the *BM* particles content from a model suspension.

For the separation tests, performed according to the method already reported by Ghimici and Constantin [14], flocs of *TMAP*_0.7_*–P*/FeO, dried for several days at room temperature and 1 day under vacuum, and a *BM* sample (MIF type -IQV, Spain) (a combination of 20% copper sulfate, slaked lime (Ca(OH)_2_), and water) have been used. Different amounts of FeO/*TMAP*_0.7_*–P* flocs were added under stirring (200 rpm) to 50 mL *BM* particle suspension placed into 100 mL beakers (c, *w*/*w*) = 0.5 g L^−1^), previously sonicated for 15 min. Stirring of the mixture was continued for a different time when the flocs amount was kept constant and 1 h for the experiments when this varied. In order to evaluate the *BM* removal percent, supernatant absorbance measurements were performed after the established settling times of the suspension. 

**Figure 10:**
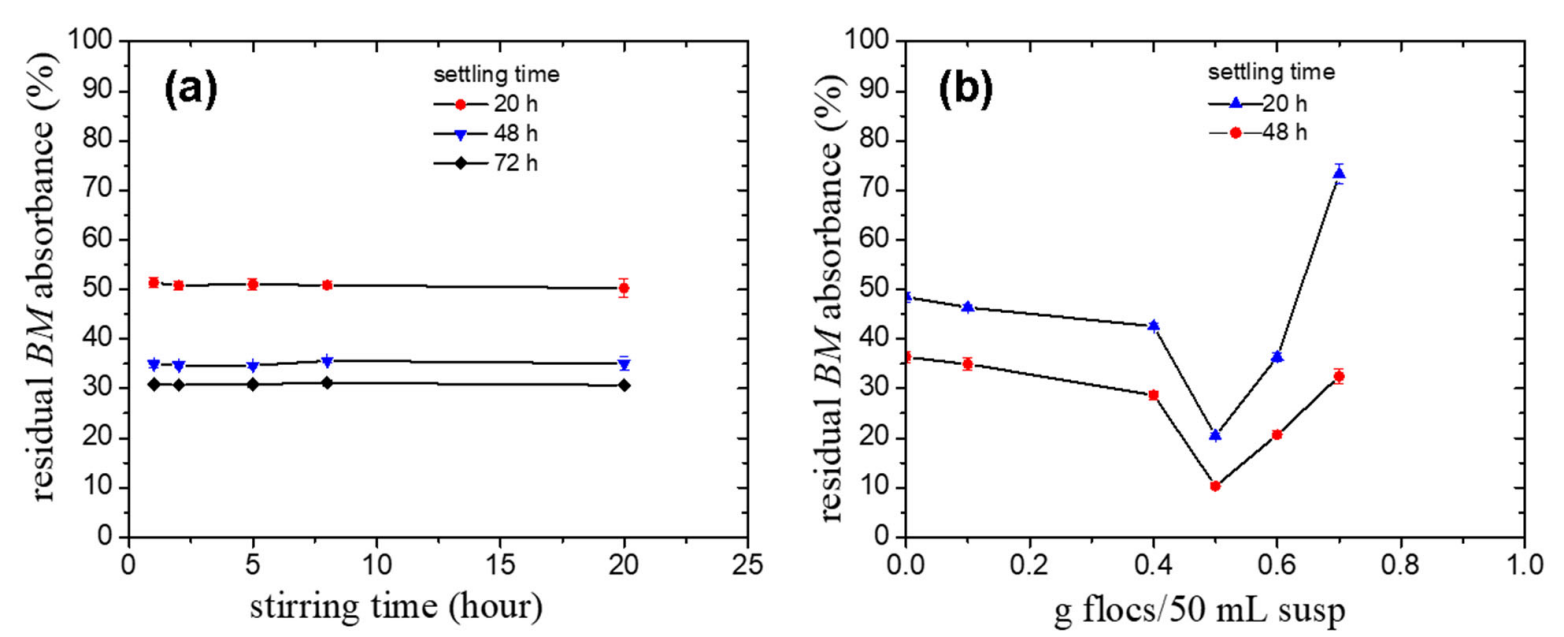
Residual *BM* absorbance (%) dependence on the stirring time (flocs dose: 0.1 g flocs/50 mL suspension) (**a**) and the flocs dose (**b**) (stirring time 1 h).

Figure 10a depicts the impact of both the stirring and settling time on the *BM* particle separation when 0.1 g flocs were used as adsorbent. The stirring time had no effect on the separation process, the residual *BM* absorbance values being constant over 20 h of stirring, at the same settling time. 

**Figure 11:**
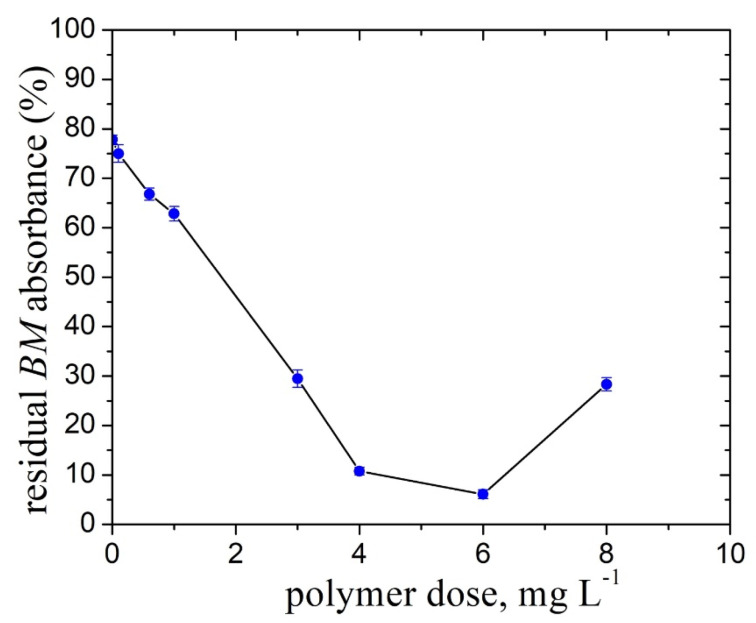
The residual *BM* absorbance (%) dependence on the polymer dose (*TMAP*_0.7_*–P*); *c*_ip_ = 1 g L^−1^; *c*_BM_ = 0.5 g L^−1^.

On the other hand, the *BM* particle removal increased with the settling time, irrespective of the period of stirring. The highest removal efficiency increase was observed after the settling time of 20 h (around 50%), with further increase in this parameter leading to an increase in removal efficiency to a lesser extent, up to 65% (48 h) and 70% (72 h). Given the finding above, Figure 10b shows only the data collected after 20 h and 48 h, for the experiments with different amounts of flocs. The flocs amount was in the range of 0.1 g/50 mL to 0.7 g/50 mL suspension of *BM*. The plotted data show a pronounced downtrend of the fungicide content in the model suspension with the flocs amount augmentation up to 0.5 g of flocs/50 mL suspension when a residual *BM* absorbance of around 10% (corresponding to a residual *BM* concentration of about 0.049 g L^−1^) was observed; the calibration curve for the *BM* suspension was not shown here. For the higher amounts of flocs, the residual *BM* absorbance values increase, which means the decline in removal efficacy of FeO/*TMAP*_0.7_*–P* flocs. In order to explain these results, we performed additional experiments where a solution of *TMAP*_0.7_*–P* or FeO particles was used for the *BM* suspension separation. As Figure 11 shows, the polymer sample has shown a very good separation efficiency for the fungicide particles, namely 94% (polymer dose 6 mg L^−1^), after 2 h settling. 

**Scheme 1:**
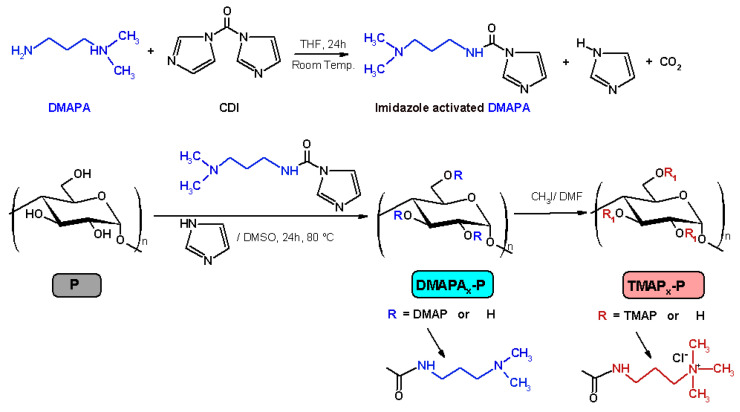
Synthesis route and chemical structure of *TMAP_x_–P* derivatives.

Regarding the implication of the FeO particles in the separation process, the tests have shown residual *BM* absorbance values around 48% (20 h settling time) and 33% (48 h settling time) for particles FeO amount/50 mL *BM* suspension between 0.01 g and 0.03 g. Given that in the absence of the metal oxide particles, the residual absorbance value for fungicide were almost the same, 49.19% (20 h settling time) and 36.33% (48 h settling time), one may assume that there are no interactions between the fungicide and the FeO particles. From these results, one may surmise that the polymer chains on the flocs surface have a decisive role in the fungicide separation process. Here, we have to remind the reader that the main flocculation mechanism implied in the FeO particle removal by the pullulan derivatives *TMAP_x_–P* is the charge patch one (see Section 2.1). It is possible that the polymer chains adsorbed on the FeO particles have free positive charges which could attract the negative species of *BM* (for example, SO_4_^2−^) (ζ = −20 mV), entailing the fungicide particle separation. Moreover, the interactions between the polymer chains and the above-mentioned contaminant could be also favored by the Cu^2+^ ions′ affinity to the amine/carbamate groups within its chemical structure (Scheme 1, Section 3.1. Materials). 

**Figure 12:**
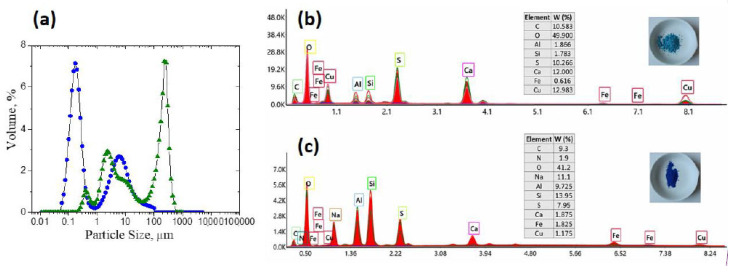
Particle size distribution for *BM* particles (blue circle) and the FeO*/TMAP*_0.7_–*P/BM* aggregates (green triangle) (**a**); EDX spectrum of the *BM* particles (**b**) and *TMAP*_0.7_–*P*/FeO*/BM* flocs (**c**). Flocs amount = 0.5 g/50 mL suspension.

Supplementary evidence for the *BM* particle retention by the *FeO/TMAP*_0.7_*–P* flocs was obtained by both particle size distribution (Figure 12a) and EDX (Figure 12b,c) measurements. 

Thus, the higher size of the flocs (D50 = 40 ± 2.36 µm) formed after the retention of *BM* particles (Figure 12a) compared to that of *TMAP*_0.7_–*P*/FeO (8.32 ± 0.39 µm, Figure 2) and the presence of Cu ions on the FeO*/TMAP*_0.7_*–P/BM* floc surface demonstrated by EDX (Figure 12c) enforce the ability of the FeO*/TMAP*_0.7_*–P* to separate BM particles from suspension. 

Although *TMAP*_0.7_*–P*/FeO flocs exhibited a slightly lower removal efficiency compared to that shown by the grafted pullulan derivatives/kreutzonit flocs, this result could give useful information regarding the beneficial use of the pullulan-based flocculants/FeO flocs for removal of the *BM* particles from simulated suspensions. We have to mention here that this study is the first step of a series of investigations where the flocs resulting from the flocculation of different inorganic particles (other metal oxides, clays) will be tested as adsorbents for other toxic organic contaminants (pesticides, dyes, etc.) from wastewater. 

## 3. Experimental

### 3.1. Materials

Cationic pullulan derivatives with three different contents of pendant quaternary ammonium groups (*TMAP_x_–P*) were prepared by a two steps reaction (Scheme 1): Firstly, pullulan (P) (Mw = 200 Kg mol^−1^ purchased from Hayashibara Lab. Ltd. (Okoyama, Japan)) was modified by the amidation reaction with 3-dimethylamino-1-propylamine (DMAPA) in dimethyl sulfoxide (DMSO) and in the presence of N,N’-carbonyldiimidazole (CDI) as activator and 4-dimethylaminopyridine (DMAP) as a catalyst, according to Constantin et al. [7,8]. Then, the pullulan derivatives containing three different amounts of tertiary amine groups (*DMAPA_x_–P*) were used as precursors in the synthesis of quaternary ammonium derivatives possessing methyl groups (*TMAP_x_–P*) [9]. Briefly, a methyl iodide solution (MeI) (18%, *w*/*v*; MeI/DMAPA molar ratio = 3/1) in DMF was dropwise added to the *DMAPA_x_–P* solution in DMF (5%, *w*/*v*) at 20 °C for 15 min. and the quaternization reaction was continued at 50 °C for 24 h. The obtained product (*TMAP_x_–P*) was precipitated in acetone. The precipitate was dissolved in 15% (*w*/*v*) NaCl solution in order to replace the iodide ions with chloride ions followed by dialysis with deionized water for 3 days to remove inorganic materials and then freeze-dried. 

Following these procedures, pullulan derivatives with various degrees of substitution (DS) with quaternary ammonium groups (number of amino groups per anhydroglucose unit in pullulan), namely 0.2, 0.4, and 0.7, were successfully obtained (chemical structure in Scheme 1). 

The polymers code is *TMAP_x_*–P, where P means pullulan, TMAP is trimethylammonium propyl carbamate group, and x = DS. 

Table 3 summarizes the synthesis parameters and some characteristics for the pullulan derivatives.

**Table 3:** Synthesis parameters and structural characteristics of synthesized pullulan derivatives.

Sample Code	P (mmol)	CDI (mmol)	DMAPA (mmol)	Molar Ratio MeI/UGU of DMAPA_x_-P	Degree of Substitution *DS* ^a^	Amine Groups Content (meq. g^−1^)
*TMAP*_0.2_ *–P*	6.17	1.54	1.54	0.56/1	0.2 ± 0.029	1.09 ± 0.108
*TMAP*_0.4_ *–P*		3.70	3.73	1.47/1	0.4 ± 0.042	2.14 ± 0.065
*TMAP*_0.7_ *–P*		6.17	6.19	2.07/1	0.7 ± 0.084	2.78 ± 0.125

^a^ represents the average value determined by ^1^H NMR and conductometric titration [7,8,9,10].

FeO particles are commercially available in packs with 150 g (Kynita SRL, Valcea, Romania). TiO_2_ particles, crystalline form anatase, were supplied by Sigma-Aldrich (Steinheim, Germany). 

NaCl, Na_2_SO_4_ (Sigma-Aldrich Chemie Gmbh, Steinheim, Germany), NaNO_3_ (Acros Organics, Geel, Germany), CaCl_2_ (Cristal R Chim SRL, Bucharest, Romania), MgCl_2_ (abcr GmbH, Karlsruhe, Germany) were used as received.

Kaolin—gift sample from Romanceram Co., Romania, chemical composition: SiO_2_, 45–55%; Al_2_O_3_, min. 34%; Fe_2_O_3_, max. 1.5%; TiO_2_, max. 0.5%; CaO and Na_2_O max. 0.25%; MgO max. 0.31%; particle size distribution: <20 μm 90–95%; <10 μm 55–65%; <6 μm 45–52%; <2 μm 30–35%; <1 μm 18–25%.

### 3.2. Methods

The simulated suspensions of metal oxide particles were prepared in distilled water, as follows: (i) FeO model dispersions: concentration (*c*_FeO_): 0.2 g L^−1^, 0.4 g L^−1^, 0.6 g L^−1^, pH = 8.34, zeta potential (ζ) = −26.7 mV; (ii) TiO_2_ model dispersions: *c*_TiO2_: 0.05 g L^−1^, pH = 5, ζ = −38.1 mV. The polymer solutions with different concentrations (*c*_ip_, g L^−1^) = 0.1, 0.3, and 1) were also prepared in distilled water and stabilized at room temperature for 1 day before use. The flocculation experiments were carried out according to the method already reported by Ghimici et al. [27]. Before starting the tests in a Cole Parmer stirrer/hotplate with 9 places, at room temperature, the dispersions of inorganic particles were sonicated for 15 min (ultrasonicator SONICS VCX 750) and then placed into 100 mL beakers (50 mL each). Different volumes of pullulan derivatives solution were added to metal oxide suspensions under stirring (500 rpm) which was kept for another 3 min. Then, the stirring speed was decreased to about 200 rpm for 15 min after which the suspension was left to settle for 120 min (FeO particles) and 1200 min (TiO_2_ ones). The above periods of settling time, called in the paper the optimum settling time (OST) (period of time after which the residual absorbance of the supernatant (%) remained almost constant) was established, in previous experiments. Subsequently, absorbance measurements (spectrophotometer SPECOL 1300 Analytik Jena, Jena, Germany) at λ = 635 nm for FeO and 400 nm for TiO_2_ particles) and zeta potential ones (Zetasizer Nano-ZS, ZEN-3500 model (Malvern Instruments, Malvern, England)) were performed on the supernatant sample (10 mL). The separation of metal oxide particles from suspension in the absence of the pullulan derivatives under the selected experimental conditions (pH, concentration of polymer and metal oxide particles, etc.) has been also evaluated. 

The metal oxides particles removal was given as a percent of the initial absorbance recorded for the inorganic particle suspensions without polymer, at time zero (residual metal oxides particles absorbance (%)):

$$\mathrm{residual}\;\mathrm{metal}\;\mathrm{oxides}\;\mathrm{particles}\;\mathrm{absorbance}\;\left(\%\right)=\frac{A_{f}}{A_{i}}\times 100$$

where, *A_i_* = the absorbance of the metal oxides suspension in the absence of polymer; and

*A_f_* = the absorbance of supernatant after the addition of the pullulan derivative.

The size distribution of the FeO particles/flocs before and after the treatment with polycations has been determined with Laser Particle Size Analyzer—Partica LA-960V2—HORIBA (Japan) (D50, µm). In addition, the surface morphology analysis was performed on FeO particles and some flocs using a Verios G4 UC Scanning Electron Microscope (Thermo Scientific, Brno, Czech Republic) equipped with energy dispersive X-ray (EDX) analyzer (Octane Elect Super SDD detector, Mahwah, NJ, USA); SEM analysis was carried out in high vacuum mode using a secondary electron detector (Everhart-Thornley Detector, Thermo Scientific, Brno, Czech Republic) at an accelerating voltage of 5 kV. 

## 4. Conclusions

The separation of FeO and TiO_2_ particles from synthetic wastewater containing the metal oxides alone or a mixture of them, as well as different salts and kaolin by cationic pullulan derivatives with different content of N,N,N-trimethylammonium propyl carbamate chloride (TMAPx–P), has been investigated. The main results of the present investigations may be outlined as follows:

- The residual metal oxides absorbance values changed with the addition of different doses of pullulan derivatives, irrespective of the particle′s dispersion medium composition.
- The *dose*_op_ values decreased with increasing ionic group content from 1 mg L^−1^. (TMAP_0.2_–P) to 0.14 mg L^−1^ (TMAP_0.7_–P) in the case of the FeO particles and from 3 mg L^−1^ (TMAP_0.2_–P) to 1.4 mg L^−1^ (TMAP_0.7_–P) in the case of the TiO_2_ ones.
- For the FeO particles removal, higher *dose*_op_ values were recorded for the initial polymer concentration, *c*_ip_ located either in the dilute regime (*c*_ip_ = 0.1 g L^−1^ − *dose*_op_= 0.5 mg L^−1^) or in the semidilute one (*c*_ip_ = 1 g L^−1^ − *dose*_op_ = 0.6 mg L^−1^) than that recorded for *c*_ip_ close to c* (*c*_ip_ = 0.5 g L^−1^ − *dose*_op_ = 0.3 mg L^−1^).
- A noticeable increase in the *dose*_op_ (0.6 mg L^−1^) and optimum dose interval (0.14 mg L^−1^ and 0.8 mg L^−1^) was found for the higher pH value than that of the natural one and the high particle suspension concentration, *c*_Feo_= 0.6 g L^−1^, respectively.
- The zeta potential and the particle aggregate size measurement results pointed to the patch flocculation mechanism.
- The pullulan derivatives proved to be good flocculants for the mixture of FeO and TiO_2_ particles (*w*/*w*, 1/1) as well as for the FeO particles from suspensions containing mixtures of salts and kaolin particles.
- The FeO*/TMAP*_0.7_*–P* flocs exhibit good performance in the removal of the fungicide Bordeaux mixture particles from simulated wastewater (residual BM content of 0.049 g L^−1^) at the optimum amount of particles (50 g flocs/1000 mL BM suspension).

## Figures and Tables

**Table 1:** Polymer-based flocculants for TiO_2_ removal.

Polymer	Mineral Oxides	*Dose*_op_	Removal Efficiency (%)	Remarks	Reference
PolyDADMAC	TiO_2_	1.25 mg polymer/g TiO_2_	-	Suspension prepared in water	[15]
Pluronic-type polymers	TiO_2_	Over the entire polymer dose	20	Suspension prepared in water	[19]
PEG		240 mg L^−1^	56		
Chitosan	TiO_2_	-	-	No flocculation in the case of the suspension prepared in distilled, but only in the tap water	[18]
Dextran derivatives	TiO_2_	1.2 ÷ 1.4 mg L^−1^	50 ÷ 60	Suspension prepared in water	[17]

**Table 2:** Ionic group content influence on *dose*_op_.

Polycation	FeO	TiO_2_
	*dose*_op_ (mg L^−1^)	
*TMAP*_0.2_ *–P*	1	3.0
*TMAP*_0.4_ *–P*	0.6	-
*TMAP*_0.7_ *–P*	0.14	1.4

## Data Availability

The data presented in this study are available on request from the corresponding author.

## References

[1] Singh R.S., Saini G.K., Kennedy J.F. (2008). Pullulan: Microbial sources, production and Applications. Carbohydr. Polym..

[2] Cheng K.C., Demirci A., Catchmark J.M. (2011). Pullulan: Biosynthesis, production, and applications. Appl. Microbiol. Biotechnol..

[3] Salehizadeh H., Yan N., Farnood R. (2018). Recent advances in polysaccharide bio-based flocculants. Biotechnol. Adv..

[4] Bataille I., Meddahi-Pelle A., Le Visage C., Letourneur D., Chaubet F., Popa V. (2011). Pullulan for biomedical uses. Polysaccharides in Medicinal and Pharmaceutical Applications.

[5] Prado H.J., Matulewicz M.C. (2014). Cationization of polysaccharides:A path to greener derivatives with many industrial applications. Eur. Polym. J..

[6] Dionísio M., Braz L., Corvo M., Lourenço J.P., Grenha A., Rosa da Costa A.M. (2016). Charged pullulan derivatives for the development of nanocarriers by polyelectrolyte complexation. Int. J. Biol. Macromol..

[7] Constantin M., Asmarandei I., Filimon A., Fundueanu G. (2015). Synthesis, characterization and solution behavior of pullulan functionalized with tertiary amino groups. High Perform. Polym..

[8] Constantin M., Bucatariu S., Ursu L., Butnaru M., Daraba O.M., Burlui A.M., Fundueanu G. (2019). Novel cationic and hydrophobic pullulan derivatives as DNA nanoparticulate carriers. Cell. Chem. Technol..

[9] Grigoras A.G., Constantin M. (2019). Solution behaviour of new pullulan derivatives with biotechnological potential. Environ. Eng. Manag. J..

[10] Moraes F.C., Antunes J.C., Forero Ramirez L.M., Aprile P., Franck G., Chauvierre C., Chaubet F., Letourneur D. (2020). Synthesis of cationic quaternized pullulan derivatives for miRNA delivery. Int. J. Pharm..

[11] Constantin M., Asmarandei I., Harabagiu V., Ghimici L., Ascenzi P., Fundueanu G. (2013). Removal of anionic dyes from aqueous solutions by an ion-exchanger based on pullulan microspheres. Carbohydr. Polym..

[12] Ghimici L., Constantin M. (2020). A review of the use of pullulan derivatives in wastewater purification. React. Funct. Polym..

[13] Ghimici L., Constantin M., Nafureanu M.-M. (2022). Grafted Pullulan Derivatives for Reducing the Content of Some Pesticides from Simulated Wastewater. Polymers.

[14] Ghimici L., Constantin M. (2021). The separation of kreutzonit particles by cationic pullulan derivatives from model suspension, *Environ*. Chall..

[15] Li D., Zhu S., Pelton R.H., Spafford M. (1999). Flocculation of dilute titanium dioxide suspensions by graft cationic polyelectrolytes. Colloid Polym. Sci..

[16] Tahir S.S., Rauf N. (2004). Removal of Fe(II) from the wastewater of a galvanized pipe manufacturing industry by adsorption onto bentonite clay. J. Environ. Manag..

[17] Ghimici L., Nichifor M. (2013). Separation of TiO_2_ particles from water and water/methanol mixtures by cationic dextran derivatives. Carbohydr. Polym..

[18] Divakaran R., Sivasankara Pillai V.N. (2004). Mechanism of kaolinite and titanium dioxide flocculation using chitosan-assistance by fulvic acids?. Water Res..

[19] Ghimici L., Brunchi C.E. (2013). Titanium dioxide separation from water by PEG and Pluronic type polymers. Sep. Purif. Technol..

[20] Ghimici L., Suflet D.M. (2015). Phosphorylated polysaccharide derivatives as efficient separation agents for zinc and ferric oxides particles from water. Sep. Purif. Technol..

[21] Tang X., Huang T., Zhang S., Wang W., Zheng H. (2020). The role of sulfonated chitosan-based flocculant in the treatment of hematite wastewater containing heavy metals. Colloids Surf. A Physicochem. Eng. Aspects.

[22] Khan J., Lin S., Nizeyimana J.C., Wu Y., Wang Q., Liu X. (2021). Removal of copper ions from wastewater via adsorption on modified hematite (α-Fe_2_O_3_) iron oxide coated sand. J. Clean. Prod..

[23] You Z., Zhao C., Sun Y., Zhuang C. (2021). Application of PAFC/CPAM for the removal of ZnO nanoparticles by enhanced coagulation. Water Sci. Technol..

[24] Bolto B., Gregory J. (2007). Organic polyelectrolytes in water treatment. Water Res..

[25] Claesson P.M., Dahlgren M.A.G., Eriksson L. (1994). Forces between polyelectrolyte-coated surfaces: Relations between surface interaction and floc properties. Colloids Surf. A Physicochem. Eng. Aspects.

[26] Petzold G., Geissler U., Smolka N., Schwarz S. (2004). Influence of humic acid on the flocculation of clay. Colloid Polym. Sci..

[27] Ghimici L., Morariu S., Nichifor M. (2009). Separation of clay suspension by ionic dextran derivatives. Sep. Purif. Technol..

[28] Bobacka V., Eklund D. (1999). The influence of charge density of cationic starch on dissolved and colloidal material from peroxide bleached thermomechanical pulp. Colloids Surf. A Physicochem. Eng. Aspects.

[29] Kleimann J., Gehin-Delval C., Auweter H., Borkovec M. (2005). Super-stoichiometric charge neutralization in particle-polyelectrolyte systems. Langmuir.

[30] Frish H.L., Simha R. (1956). The Viscosity of Colloidal Suspensions and Macromolecular Solution.

[31] Wolf B.A. (2007). Polyelectrolytes revisited: Reliable determination of intrinsic viscosity. Macromol. Rapid Commun..

[32] Smith K.M., Fowler G.D., Pullket S., Graham N.J.D. (2009). Sewage sludge-based adsorbents: A review of their production, properties and use in water treatment applications. Water Res..

[33] Roy M.M., Dutta A., Corscadden K., Havard P., Dickie L. (2011). Review of biosolids managements options and co-incineration of a biosolid-derived fuel. Waste Manag..

[34] Gianico A., Braguglia C.M., Gallipoli A., Montecchio D., Mininni G. (2021). Land Application of Biosolids in Europe: Possibilities, Con-Straints and Future Perspectives. Water.

[35] Hanum F., Yuan L.C., Kamahara H., Abdul Aziz H., Atsuta Y., Yamada T., Daimon H. (2019). Treatment of Sewage Sludge Using Anaerobic Digestion in Malaysia: Current State and Challenges. Front. Energy Res..

[36] Babatunde A.O., Zhao Y.Q. (2007). Constructive Approaches Toward Water Treatment Works Sludge Management: An International Review of Beneficial Reuses. Environm. Sci. Technol..

[37] Devi P., Saroha A.K. (2017). Utilization of sludge based adsorbents for the removal of various pollutants: A review. Sci. Total Environ..

[38] Soliman N.K., Moustafa A.F. (2020). Industrial solid waste for heavy metals adsorption features and challenges; a review. J. Mater. Res. Technol..

[39] Guerra-Rodríguez S., Oulego P., Rodríguez E., Singh D.N., Rodríguez-Chueca J. (2020). Towards the Implementation of Circular Economy in the Wastewater Sector: Challenges and Opportunities. Water.

[40] Taheriyoun M., Memaripour A., Nazari-Sharabian M. (2020). Using recycled chemical sludge as a coagulant aid in chemical wastewater treatment in Mobarakeh Steel Complex. J. Mater. Cycles Waste Manag..

[41] Gupta V.K., Ali I., Suhas S.V.K. (2006). Adsorption of 2,4-D and carbofuran pesticides using fertilizer and steel industry wastes. J. Colloid Interface Sci..

[42] Hu Y.S., Zhao Y.Q., Sorohan B. (2011). Removal of glyphosate from aqueous environment by adsorption using water industrial residual. Desalination.

[43] Sousa S., Jiménez-Guerrero P., Ruiz A., Ratola N., Alves A. (2011). Organochlorine pesticides removal from wastewater by pine bark adsorption after activated sludge treatment. Environ. Technol..

